# Single-read tRNA-seq analysis reveals coordination of tRNA modification and aminoacylation and fragmentation

**DOI:** 10.1093/nar/gkac1185

**Published:** 2022-12-20

**Authors:** Xavier Hernandez-Alias, Christopher D Katanski, Wen Zhang, Mahdi Assari, Christopher P Watkins, Martin H Schaefer, Luis Serrano, Tao Pan

**Affiliations:** Centre for Genomic Regulation (CRG), The Barcelona Institute of Science and Technology, Barcelona 08003, Spain; Department of Biochemistry and Molecular Biology, University of Chicago, Chicago, IL 60637, USA; Department of Biochemistry and Molecular Biology, University of Chicago, Chicago, IL 60637, USA; Department of Chemistry, University of Chicago, Chicago, IL 60637, USA; Department of Biochemistry and Molecular Biology, University of Chicago, Chicago, IL 60637, USA; IEO European Institute of Oncology IRCCS, Department of Experimental Oncology, Milan 20139, Italy; Centre for Genomic Regulation (CRG), The Barcelona Institute of Science and Technology, Barcelona 08003, Spain; Universitat Pompeu Fabra (UPF), Barcelona 08002, Spain; ICREA, Pg. Lluís Companys 23, Barcelona 08010, Spain; Department of Biochemistry and Molecular Biology, University of Chicago, Chicago, IL 60637, USA

## Abstract

Transfer RNA (tRNA) utilizes multiple properties of abundance, modification, and aminoacylation in translational regulation. These properties were typically studied one-by-one; however, recent advance in high throughput tRNA sequencing enables their simultaneous assessment in the same sequencing data. How these properties are coordinated at the transcriptome level is an open question. Here, we develop a single-read tRNA analysis pipeline that takes advantage of the pseudo single-molecule nature of tRNA sequencing in NGS libraries. tRNAs are short enough that a single NGS read can represent one tRNA molecule, and can simultaneously report on the status of multiple modifications, aminoacylation, and fragmentation of each molecule. We find correlations among modification-modification, modification-aminoacylation and modification-fragmentation. We identify interdependencies among one of the most common tRNA modifications, m^1^A58, as coordinators of tissue-specific gene expression. Our method, **S**ing**L**e-read **A**nalysis of **C**rosstalks (SLAC), reveals tRNAome-wide networks of modifications, aminoacylation, and fragmentation. We observe changes of these networks under different stresses, and assign a function for tRNA modification in translational regulation and fragment biogenesis. SLAC leverages the richness of the tRNA-seq data and provides new insights on the coordination of tRNA properties.

## INTRODUCTION

Transfer RNAs are highly abundant non-coding RNAs of 65–94 nucleotides, which form a rigid secondary structure. Up to 20% of all residues in an eukaryotic tRNA are modified (1). tRNAs are aminoacylated (charged) with an amino acid at the 3′ end. Charged tRNAs are required for protein synthesis by recognizing their cognate codons in the elongating ribosomes (2). The fine-tuned regulation of tRNA abundance, modification and charging determines the structure and function of tRNA in translation, and their alterations can lead to human diseases including neurological disorders and cancer (1,3,4).

All three properties of the cellular tRNAome are highly dynamic and respond to development and environmental cues (5,6). tRNA abundance is tissue-specific in humans and reprogrammed in cancers that adapt to the codon usage changes of the tissues and cancer phenotypes (4,7). tRNA charging levels are sensitive to stress conditions; its response to amino acid starvation regulates selective translation of stress-response genes and tunes global translation activity through phosphorylation of eIF2α by the GCN2 kinase (3,8). Dynamic response of tRNA modifications occurs at many modification types and sites which fine-tune translation of stress response genes in a codon-dependent manner, as well as the biogenesis of tRNA fragments that are functional small RNAs in the regulation of mRNA stability and cell-cell communication (1,2).

The advent of high throughput tRNA sequencing (9–12) allows for the examination of tRNA abundance, modification and charging in the same sequencing library. NGS sequencing requires cDNA synthesis of the RNA by reverse transcription. Certain tRNA modifications that perturb the Watson–Crick base pairing such as N1-methyladenosine (m^1^A) do not always stop reverse transcription, rather, some reverse transcriptases can readthrough these modifications while leaving behind a ‘mutation’ signature relative to the tRNA reference sequence in data analysis (10,13,14). All mature tRNAs end with 3′CCA. The aminoacylated tRNA levels can be measured upon incorporating a periodate oxidation and beta-elimination step in the sequencing library preparation; periodate only oxidizes uncharged tRNAs and the oxidized 3′ A residue is removed upon beta-elimination. Therefore, uncharged tRNA ends with 3′CC and charged tRNA ends with 3′CCA, and the aminoacylation levels for each tRNA can be measured by the ratio of their 3′CCA and 3′CC ending reads (9,10).

A human tRNA contains on average 13 modifications per molecule, how these modifications are linked to each other in their installation and function is currently under intense investigation (15). For example, the installation of multiple anticodon loop modifications in yeast tRNA^Phe^ exhibits a specific order (16). Q and m^5^C modifications in *Schizosaccharomyces pombe* tRNA^Asp^ depend on one another (17). Such studies so far have mostly focused on the multiple anticodon loop modifications that are all located in the same hairpin loop (18–20). How modifications that are distal in the tRNA secondary structure are dependent on each other remains an open question. In addition, very little is known about the potential crosstalks between tRNA modification-modification and tRNA charging-modification (21).

Here, we implement SLAC (**S**ing**L**e-read **A**nalysis of **C**rosstalks), a computational pipeline to investigate the crosstalk between tRNA modifications, aminoacylation and fragmentation transcriptome-wide. SLAC is a single-read analysis pipeline of tRNA-sequencing data that takes advantage of recent advances in read-length, charging, modification, and fragment detection in tRNA. We show that correlating modification-induced mutation signatures with aminoacylation measurements at the single-read level confirms known crosstalks in yeast, and identifies new crosstalks in human tRNAs. We identify tissue-specific crosstalk signatures in mice. We observe stress-response changes in modification and charging that are consistent with these crosstalks. We show that tRNA modification and fragmentation are associated in a tRNA-type and cleavage-site dependent manner. Our results support the notion that tRNA modification and charging crosstalks may play a previously under-appreciated role in translational regulation.

## MATERIALS AND METHODS

### Data sources

#### tRNA-seq datasets

The raw FASTQ files of mim-tRNA-seq of *Saccharomyces cerevisiae* and of HEK293T cells were retrieved from Gene Expression Omnibus (GEO): GSE152621 (10). In this dataset, all HEK293T samples, three WT yeast samples (‘WT’ in Supplementary Table S2) and the *Trm7Δ* mutant are periodate oxidized, and therefore both aminoacylation and modification levels are detectable. However, three WT yeast samples (‘WTox0’ in Supplementary Table S2), and *Trm1Δ* and *Trm10Δ* mutants are not periodate oxidized and only modification levels are detectable.

The raw FASTQ files of QuantM-tRNA-seq of mouse tissues and of HEK293T cells were retrieved from GEO: GSE141436 (14). The raw FASTQ files of Charged DM-tRNA-seq of HEK293T cells were retrieved from GEO: GSE97259 (9). The raw FASTQ files of total and polysome MSR-seq data of HEK293T in control and stress were retrieved from GEO: GSE198441 (12).

#### Coding sequences and expression

The mRNA-seq gene expression data of total and polysome HEK293T in control and stress were directly downloaded from Watkins *et al.* (12). The coding sequences of all human transcripts were computed from the same reference transcriptome.

### Computational analysis

#### Read preprocessing

For QuantM-tRNA-seq data, raw FASTQ reads were first trimmed using Seqtk (github.com/lh3/seqtk, v1.3) and then 3′ and 5′ adaptors removed with BBDuk (sourceforge.net/projects/bbmap, v38.79), as described in their original protocol (14). For MSR-seq data, raw FASTQ reads were adaptor-removed using BBMerge (22) (sourceforge.net/projects/bbmap, v38.79) with: mininsert = 20, mininsert0 = 20, trimpolya = t, usequality = f, forcemerge = t, entropy = f, adapter1 = GATCGTCGGACTGTAGAA, adapter2 = AGATCGGAAGAGCACACGTCTGAACTCCAGTCAC. Then 3′ and 5′ ends were trimmed to remove the barcode and library-added nucleotides using Seqtk -b 7 -e 6 (github.com/lh3/seqtk, v1.3). For DM-tRNA-seq data, raw FASTQ reads were adaptor-removed using BBMerge (22) (sourceforge.net/projects/bbmap, v38.79) with: mininsert = 20, mininsert0 = 20, trimpolya = t, usequality = f, forcemerge = t, entropy = f, adapters = AGATCGGAAGAGCACACGTCTGAACTCCAGTCA. Then RT adapters were trimmed from 3′-ends with BBDuk (sourceforge.net/projects/bbmap, v38.79): literal = CTTTGAGCCTAATGCCTGAAAGATCGGAAGAGCACACGTCTAGTTCTACAGTCCGACGATC, mink = 8, ktrim = r, k = 10, hdist = 1, minlength = 10.

#### tRNA quantitation and analysis

Quantitation and analysis of tRNA expression, charging and modifications were performed using the mim-tRNA-seq computational pipeline (10) (github.com/nedialkova-lab/mim-tRNAseq, v0.3.4.5). The following parameters were used:

*S. cerevisiae*: –species Scer –cluster-id 0.90 –min-cov 0.0005 –max-mismatches 0.1 –control-condition WT –max-multi 4 –remap –remap-mismatches 0.075

*M. musculus*: –species Mmus –cluster-id 0.95 –min-cov 0.0005 –max-mismatches 0.1 –control-condition Liver –max-multi 4 –remap –remap-mismatches 0.075

*H. sapiens*: –species Hsap –cluster-id 0.95 –min-cov 0.0005 –max-mismatches 0.1 –control-condition control_total –max-multi 4 –remap –remap-mismatches 0.075

#### Single-read analysis

After read deconvolution of the mim-tRNA-seq computational pipeline (10), all mismatches and charging of each read were recorded in a tab-delimited format. For all positions with at least 5% mismatch rate, the single-read analysis was performed. For each pair of either two modified positions or modification and charging, the algorithm computed the odds ratio (OR).

$$\begin{equation*}OR = \frac{(\sharp\, reads\ with\ both\ A\ and\ B ) \times ( \sharp\, reads\ without\ A\ nor\ B )}{( \sharp\, reads\ with\ A,\ but\ not\ B ) \times (\sharp\, reads\ with\ B,\ but\ not\ A )}\end{equation*}$$

The significance of the crosstalk was determined by Fisher's exact test. Finally, *P*-values were FDR-corrected for multiple comparisons with Benjamini & Hochberg.

#### tRNA fragmentation crosstalks

For fragmentation analysis, only 5' tRFs were considered, since 3' tRFs cannot be distinguished from RT stops. tRNA reads were classified in four different classes based on their 3' end: tRFs terminating at positions 30–39 (C-loop), tRFs at 40–49 (V-loop), tRFs at 50–59 (T-loop), and full-length tRNAs longer than position 60. Similar to the single-read analysis above, we computed the odds ratio between all positions with at least 5% mismatch rate and each of the three tRF classes versus full length tRNAs.

$$\begin{equation*}OR= \frac{( \sharp\, tRX\ reads\ with\ modification ) \times ( \sharp\, Full\ tRNA\ reads\ without\ modification )}{( {\sharp\, tRX\ reads\ without\ modification} ) \times ( \sharp\, Full\ tRNA\ reads\ with\ modification)}\end{equation*}$$

The significance of the crosstalk was determined by Fisher's exact test. Finally, *p*-values were FDR-corrected for multiple comparisons with Benjamini and Hochberg.

### Simulated data

To validate the method against an artificially generated dataset, we generated samples containing 20 000 full-length yeast tRNA^Phe^(GAA)-2 reads each. Mismatches were incorporated into positions 26 and 58 of the sequence with a certain probability (*p*_A_, *p*_B_). Given a certain OR, the probability of having both modifications in the same read was determined as:

$$\begin{equation*}{p}_{AB}\ = \frac{{1 + \left( {{p}_A + {p}_B} \right)\left( {OR - 1} \right) - \sqrt {{{\left( {1 + \left( {{p}_A + {p}_B} \right)\left( {OR - 1} \right)} \right)}}^2 + 4 \cdot OR\left( {1 - OR} \right) \cdot {p}_A \cdot {p}_B} }}{{2\left( {OR - 1} \right)}}\end{equation*}$$

A total of 700 samples were generated with all combinations of *p*_A_,*p*_B_ = [0.075, 0.175, 0.275, 0.375, 0.475, 0.575, 0.675, 0.775, 0.875, 0.975] and log_2_(OR) = [–1.0, –0.5, –0.25, 0.0, 0.25, 0.5, 1.0]. They were finally aligned and analyzed by SLAC.

#### Differential modification and aminoacylation analysis

To test the significance of changes in modification between conditions, we first computed the counts of reads with or without mismatch at modified positions (>5% absolute mismatch rate). For changes in aminoacylation in periodate-treated samples, we counted the reads ending with CCA or CC. Next, a contingency table was built with the counts of modified/unmodified or charged/uncharged reads in condition A and condition B, and the odds ratios were calculated as:

$$\begin{equation*}OR= \frac{{\left( {\sharp\, reads\ modified/charged\ in\ A} \right)/\left( {\sharp\, reads\ unmodified/uncharged\ in\ A} \right)}}{{\left( {\sharp\, reads\ modified/charged\ in\ B} \right)/\left( {\sharp\, reads\ unmodified/uncharged\ in\ B} \right)}}\end{equation*}$$

The significance of each OR was determined by chi-square tests. Finally, *p*-values were FDR-corrected for multiple comparisons with Benjamini and Hochberg.

#### Translational efficiency analysis

Using the human mRNA-seq data of control and stressed HEK293T cells, we computed the translational efficiency (TE) as the ratio between polysome TPMs versus total RNA TPMs, taking only genes with at least 10 TPM in both datasets.

#### Relative synonymous codon usage (RSCU)

The RSCU is defined as the ratio of the observed frequency of a certain codon to the expected frequency given that all the synonymous codons for the same amino acid were used equally. The RSCU is therefore a real value between 0 and the number of synonymous codons for that amino acid, with values <1 indicating a lower observed usage than expected, and vice versa.

$$\begin{equation*}RSCU\ = \frac{{{x}_C}}{{\mathop \sum \nolimits_{i \in {C}_{aa}}^{{n}_{aa}} {x}_i}}\ \times {n}_{aa}\end{equation*}$$

where ${x}_C$ refers to the abundance of the codon $C$, ${C}_{aa}$ is the set of all synonymous codons, and ${n}_{aa}$ is the number of synonymous codons.

To determine the RSCU of a specific condition, we computed the average of the RSCU of all genes weighted by their standardized log_2_(TE).

#### Statistical analysis

All details of the statistical analyses can be found in the text and figure legends. We used a significance level of 0.05 for all analyses. Significant differences are abbreviated as follows: ns (*P* > 0.05), * (*P* ≤ 0.05), ** (*P* ≤ 0.01), *** (*P* ≤ 0.001) and **** (*P* ≤ 0.0001).

## RESULTS

### Single-read tRNA-seq analysis reveals known and new crosstalks in yeast tRNA^Phe^

**Figure 1.**
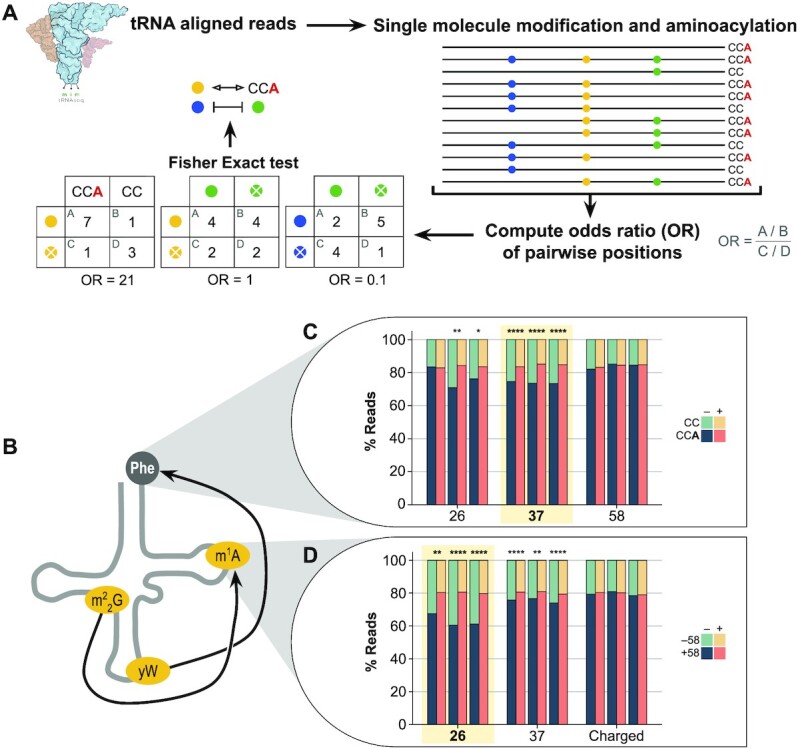
Single-read tRNA-seq analysis reveals known and new crosstalks in yeast tRNA^Phe^. (**A**) Schematic pipeline for the single-read analysis of tRNA reads. OR: odds ratio. (**B**) Detected known crosstalks of tRNA^Phe^ of yW37-Charging and m^2^_2_G26-m^1^A58 that are highlighted in (C) and (D). (**C**) Percentage of charged tRNA reads with possible crosstalks with the m^2^_2_G26, yW37, and m^1^A58 modifications. (**D**) Percentage of m^1^A58-modified reads with possible crosstalks with the m^2^_2_G26, yW37 and charging. In (C) and (D), the biological triplicates are plotted separately. Significant crosstalks are indicated with * (*P* < 0.05), ** (*P* < 0.01), *** (*P* < 10^−3^), **** (*P* < 10^−4^). Significance is determined by Fisher's exact test, and FDR-corrected for multiple comparisons with Benjamini and Hochberg.

Because of its size, tRNA-seq produces reads that can cover the entire length of the tRNA; tRNA-seq also captures certain tRNA modifications as ‘mutations’ relative to the reference tRNA sequence, and depending on the library construction protocol the charging status by the heterogeneous 3′ ends in the sequencing data (9,10,12). On average, one of every four modifications leave a mismatch signature upon reverse transcription, which makes them detectable by tRNA-seq (Supplementary Table S1). However, the analysis of modifications and charging is generally reduced to a simple pileup percentage, which does not take into account the single-read nature of the data. In fact, previous reports using Sanger sequencing have provided proof-of-concept evidence that modification signatures can be quantified and correlated at the single-read level to detect crosstalks (23). In SLAC, we consider all pairwise combinations of tRNA modified positions (i.e. positions with at least 5% reads containing ‘mutations’) and charging, as well as all pairwise combinations of two modified positions that are detectable through ‘mutations’ (Figure 1A). For charging and modification crosstalks, we determine the number of reads for: (i) tRNA is charged and modified, (ii) tRNA is charged but not modified, (iii) tRNA is not charged but modified and (iv) tRNA is not charged and not modified. For any two modification crosstalks, we determine the number of reads for: (i) both sites are modified, (ii) site 1 is, site 2 is not modified, (iii) site 1 is not, site 2 is modified and (iv) both sites are not modified.

Our analysis produces an odds ratio (OR) that informs whether tRNA charging and modification or any pair of modifications tend to appear together in the same read (OR > 1, stimulatory crosstalk) or tend to be exclusive of one another (OR < 1, inhibitory crosstalk), as well as calculates the significance of this interdependence using Fisher's exact test. Given that the tRNA-seq data in general has very high coverage for each tRNA species, hundreds to thousands of pairs for a specific tRNA can be analyzed simultaneously and the resulting p-values are FDR-corrected for multiple comparisons. We implemented our method within the open-source mim-tRNA-seq computational pipeline (10) (https://github.com/nedialkova-lab/mim-tRNAseq).

To validate our method, we analyzed yeast mim-tRNA-seq data from Behrens *et al.* (10), since most prior knowledge on tRNA crosstalks was centered on yeast tRNA^Phe^ (15) (Supplementary Table S2). Among all modifications detectable by tRNA-seq (Figure 1B, Supplementary Figure S1A), there were two known crosstalks against which our method could be tested: the interdependence between wybutosine (yW) at position 37 (all residue numbering are according to the standard tRNA nomenclature, 37 corresponds to the 3′ immediate nucleotide after the anticodon) and charging (21) and the association between N2,2-dimethyl-G (m^2^_2_G) at position 26 (in between the D and anticodon stems) and m^1^A at position 58 (in T loop) (16). Our single-read pipeline identified both crosstalks in all three biological replicates (Figure 1C-D). Furthermore, we identified interdependencies (yW37-m^1^A58, m^2^_2_G26-Charging) that were previously unknown. Apart from tRNA^Phe^, we detected crosstalks expanding to other tRNA species in wild-type yeast cells (Supplementary Figure S1B, Supplementary Table S2).

Although SLAC detects statistical association between two modifications or modification-charging, the method inherently lacks causality or directionality. Some of the detected crosstalks may be causal, i.e. they represent coordinated actions of tRNA acting enzymes, whereas others may be derived from indirect or independent events. Evidence of causality may be established through tRNA acting gene knockout experiments. We analyzed mim-tRNA-seq data of yeast strains deficient of modification enzyme *Trm7* (lacking yW37 in tRNA^Phe^(GAA), Supplementary Figure S1C, left panel) (10). tRNA^Phe^ has known crosstalks between 37 and charging (Figure 1C). Indeed, *Trm7Δ* led to a significant decrease in charging, consistent with yW37 modification enhancing tRNA^Phe^ charging.

We also analyzed data from yeast strains with deletions of *Trm1* (lacking m^2^_2_G26, Supplementary Figure S1C, middle panel) and *Trm10* (lacking m^1^G9, Supplementary Figure S1C, right panel) (10). In contrast to *Trm7Δ*, the *Trm1Δ* and *Trm10Δ* samples were not treated with periodate in library construction and hence only modification crosstalks could be examined. We established that the gene knockouts led to large decreases in the mutation fractions of the corresponding positions in the known tRNA substrates as expected, 37 for *Trm7Δ*, 26 for *Trm10Δ* and 9 for *Trm10Δ* (Supplementary Figure S1C, Supplementary Table S2). Among the previously identified crosstalks (Supplementary Figure S1B), *Trm1Δ* increased the m^1^G9 level of tRNA^Lys^(CTT) and decreased m^1^A58 level of tRNA^Lys^(CTT), tRNA^Phe^(GAA) and tRNA^Thr^(TGT). These results suggest that m^2^_2_G26 partially inhibits m^1^G9 modification as well as enhances m^1^A58 modification in these tRNAs.

We also found some modification crosstalks present in the wild-type samples that did not respond or responded differently to the modification enzyme knockouts, or new crosstalks only present in the enzyme knockout samples. The former includes 26–37, 37–58 in *Trm7Δ*; 26–32, 26–37, several 26–58 in *Trm1Δ*; and 9–26, 9–32 and 9–58 in *Trm10Δ*. These may represent crosstalks that are either unidirectional, e.g. m^2^_2_G26-m^1^G9 crosstalk of tRNA^Lys^(CTT) in *Trm1Δ* but not in *Trm10Δ*, or derived from multiple or independent events rather than coordinated action of two specific modification enzymes. The latter, such as three 9–26 in *Trm1Δ* and two 9–58 in *Trm10Δ*, may represent ‘synthetic’ crosstalks that become only prevalent upon the knockout of a specific modification enzyme, akin to the synthetic phenotypes in genetics that become observable only upon the deletion of a specific gene. Therefore, while all known crosstalks in the literature are recovered by SLAC (Figure 1C-D), not all detected crosstalks by SLAC change in the same way in every tRNA upon modification enzyme perturbation.

We further validated the sensitivity of the method by generating simulated reads with different odds ratios (see Methods) and analyzing them with SLAC. We found that we can detect with confidence odds ratios as low as 1.20 (i.e. log_2_(OR) = 0.25), especially for mutation fractions in the sequencing data ranging between 5–95% (Supplementary Figure S1D). These results indicate that tRNA-seq data can be used at single-read level to retrieve interdependence information between charging and modification, and between two modified positions.

### m^1^A58-related crosstalks are abundant in the human tRNAome

**Figure 2.**
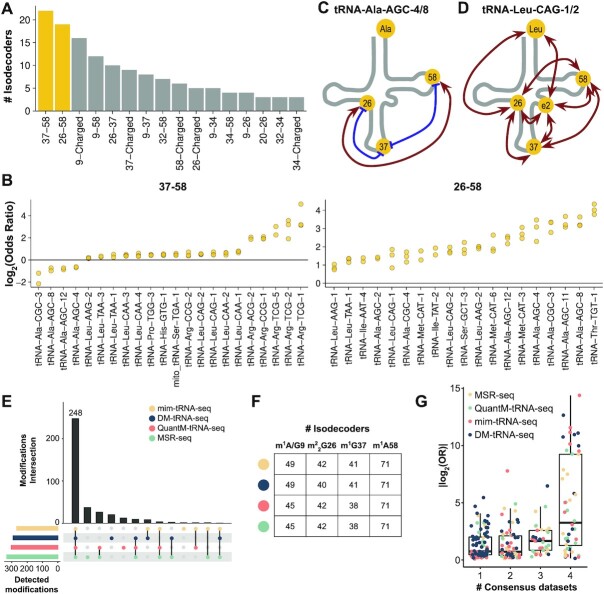
m^1^A58-related crosstalks are abundant in the human tRNAome. (**A**) Histogram of all significant crosstalks in at least two of the three biological replicates of human tRNA in HEK293T cells. Only crosstalks detected in three or more tRNA isodecoders are shown. Known modifications that generate high levels of mutation signatures in this sequencing experiment are m^1^G9, acp^3^U20, m^2^_2_G26, m^3^C32, I34, m^1^I37/m^1^G37 and m^1^A58. (**B**) Distribution of OR of detected 37–58 and 26–58 pairs, with each dot corresponding to individual replicates. (**C**, **D**) Significant crosstalks detected in at least two of the three biological replicates of tRNA^Ala^(AGC)-4/8 and tRNA^Leu^(CAG)-1/2. Position e2 is a m^3^C modification. (**E**) Upset plot of detected modification sites (>5% mismatch in read alignment) in all replicates of HEK293T cells by mim-tRNA-seq, DM-tRNA-seq, QuantM-tRNA-seq, and MSR-seq. (**F**) Number of isodecoders with detected m^1^A/G9, m^2^_2_G26, m^1^G37 and m^1^A58 modifications in all replicates of HEK293T cells by mim-tRNA-seq, DM-tRNA-seq, QuantM-tRNA-seq, and MSR-seq. (**G**) Absolute log_2_-transformed odds ratios of crosstalks detected in HEK293T cells by 1, 2, 3 or 4 methods. The residue numbers for each tRNA is according to the tRNA nomenclature, e.g. the wobble anticodon nucleotide is always 34. tRNA transcript label is according to the genomic tRNA database (40).

We next analyzed the DM-tRNA-seq dataset of human HEK293T cells which also measured charging (9) (Supplementary Table S3). Among all detected interdependencies, we identified m^1^A58 crosstalks with positions 26 and 37 among the most frequent, present in 22 and 19 different tRNA isodecoders, respectively (Figure 2A). By analyzing the OR of the 37–58 pair (Figure 2B), we found that the m^1^A58 modification is generally positively correlated with m^1^G37/m^1^I37 (OR > 1), except in tRNA^Ala^ isodecoders where the m^1^A58 modification has an inhibitory effect on m^1^I37 (OR < 1). On the other hand, m^2^_2_G26 appears always positively correlated with m^1^A58. Moreover, most crosstalks do not occur alone, rather they coordinate with other pairs within the same tRNA molecule. Therefore, specific tRNAs form intricate interdependent networks of multiple modified positions and charging. For instance, the isodecoders tRNA^Ala^(AGC)-4/8 exhibit a network among three modification sites (Figure 2C) involving m^2^_2_G26, m^1^I37 and m^1^A58. In tRNA^Leu^(CAG)-1/2 (Figure 2D), the network is even more complex with a total of eight significant crosstalks among m^2^_2_G26, m^1^G37, m^3^C-e2 (in the loop of the variable hairpin loop in type II tRNA), m^1^A58, and charging. Other isodecoders show simpler networks with just one or few detected crosstalks (Supplementary Figure S2A).

For pairwise correlation of modifications we benchmarked SLAC against published QuantM-tRNA-seq, mim-tRNA-seq, DM-tRNA-seq and MSR-seq methods where datasets of HEK293T cells were available (9,10,12,14) (Supplementary Table S3). In the detection of the mutation signatures, MSR-seq and QuantM-tRNA-seq used the SuperScript IV (SSIV) reverse transcriptase while mim-tRNA-seq and DM-tRNA-seq employed TGIRT that have similar tendencies to generate mutation signature in the tRNA-seq data. We detected a good degree of concordance, with a total of 248 consensus modifications identified by mutation signatures among all 380 mutated positions identified (>65%, Figures 2E, F). However, at the quantitative level, modification signatures of TGIRT-based methods are clearly different from SSIV-based methods (Supplementary Figure S2B, C). As a result of these protocol differences and the distinct depth of reads coverage, we observed many common crosstalks as well as others that were dependent on the tRNA-seq dataset, which expanded our initial set of detected crosstalks (Supplementary Figure S2E, Supplementary Table S3). A more detailed analysis actually showed that the consensus detected crosstalks between datasets are generally pairs with more extreme OR (Figure 2G). Altogether, our exploration of the HEK293T human tRNAome shows a high interconnectivity among modifications and between modifications and charging, often involving m^1^A58.

### Tissue-specificity of m^1^A58 and crosstalks across mouse tissues

**Figure 3.**
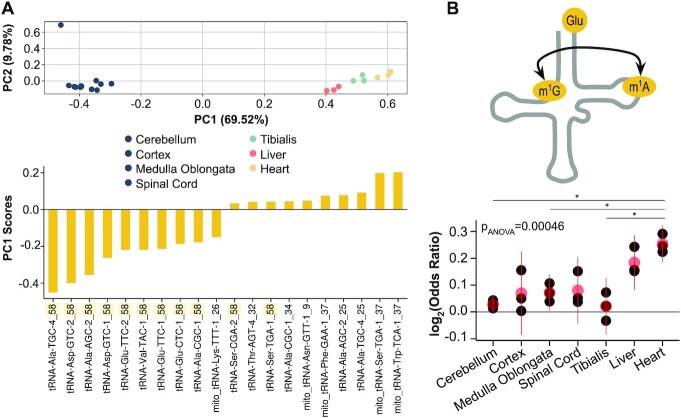
Tissue-specificity of m^1^A58 and crosstalks across mouse tissues. (**A**) Principal component analysis (PCA) of all tRNA modifications across seven mouse tissues (top), and the respective top 10 positive and negative contributors of the first component (bottom). (**B**) Changes in OR among tissues of tRNA^Glu^(TTC)-1. Black dots are individual replicates, red dots and lines indicate the mean ± SD. Significance is determined with ANOVA and post-hoc Student's *t*-test, FDR-corrected for multiple comparisons with Benjamini and Hochberg.

We next characterized the relevance of tRNA modifications and their crosstalks beyond cell cultures using the publicly available QuantM-tRNA-seq data of seven mouse tissues (14) (Supplementary Table S4). The m^1^A58 modification is among the most widespread tRNA modifications in mammals, is present in the T loop of almost all cytosolic tRNAs, and can be detected at high sensitivity through mutation signatures in tRNA-seq (11,24). As previously reported, the mutation fraction derived from m^1^A58 has a highly tissue-specific pattern (Supplementary Figure S3). A principal component analysis (PCA) of all modifications can clearly discriminate four main clusters of samples corresponding to brain (containing cortex, spinal cord, medulla oblongata and cerebellum; without clear differences among them), liver, tibialis, and heart tissues (Figure 3A). The first component, which explains almost 70% of the variance, is predominantly determined by the m^1^A58 fraction of several isodecoders among tRNA^Ala^, tRNA^Asp^ and tRNA^Glu^ (Figure 3A). On the other hand, the second component, which explains <10% of the variance, is more related to the technical variability of mismatches, which is particularly evident for one biological replicate of the cortex. We also observe a significant tissue-specificity of the interdependence between m^1^A58 and m^1^G9 in tRNA^Glu^(TTC)-1 (ANOVA, *P* < 0.05) (Figure 3B), with liver and heart tissues showing a higher concurrence of both modifications. Overall, the mouse tissue data indicates a tissue-specific nature of the m^1^A58 modification and its related crosstalks.

### Crosstalks recapitulate modification and charging changes upon stress to potentially regulate translation

**Figure 4.**
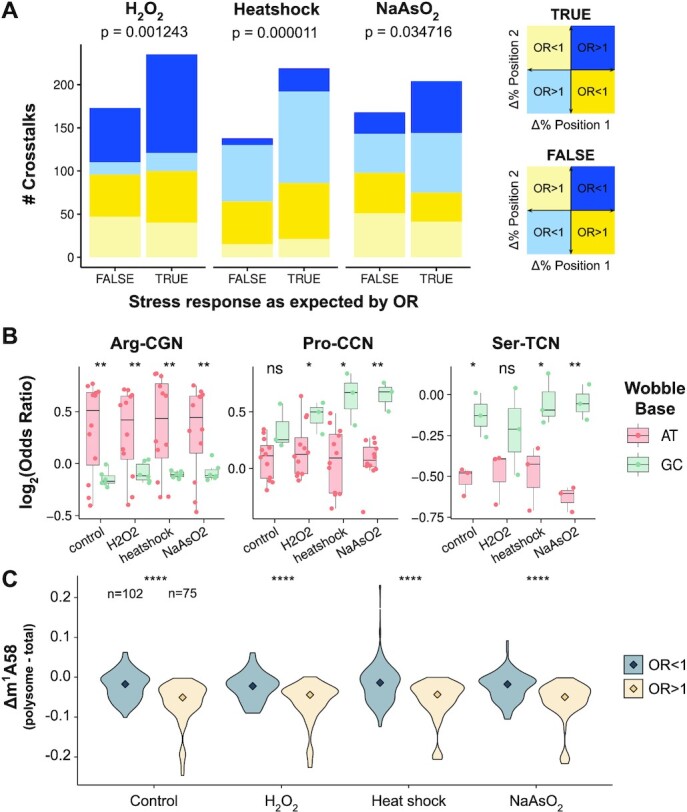
Crosstalks recapitulate modification and charging changes upon stress to potentially regulate translation. (**A**) Number of position pairs changing in stress as expected by SLAC crosstalks (TRUE) or not (FALSE), related to Supplementary Figure S4A. All significant crosstalks of >3% changes in mismatch in read alignment are included. A one-sided binomial test is used to determine whether the observed frequency of successes is significantly higher than the null model *p* = 0.5. (**B**) Differences in OR of m^1^A58-charged between isodecoders having AT versus GC at the wobble position for 4-codon-box readers of tRNA^Arg^, tRNA^Pro^ and tRNA^Ser^. Significance is determined by a two-sided Student's *t*-test: * (*P* < 0.05), ** (*P* < 0.01), *** (*P* < 10^−3^), **** (*P* < 10^−4^). (**C**) Differences in m^1^A58 modification between polysome versus total tRNA among isodecoders with OR > 1 and OR < 1. Changes between tRNAs with OR > 1 and OR < 1 are detected by two-tailed Wilcoxon rank-sum test (**** *P* < 10^−4^).

To investigate the regulatory potential of tRNA modification and charging crosstalks in stress response, we analyzed the MSR-seq data of HEK293T cells upon three different stresses: heat shock, hydrogen peroxide, and arsenite, which have been shown to alter tRNA properties that regulate translation (12). The MSR-seq data contains tRNA abundance, modification and charging measurements and mRNA measurements for both total RNA and polysome associated RNA, so that the effect on translational response under stress can be readily examined. For X–Y crosstalks with OR > 1, we hypothesize a perturbation that causes an increase in X would also induce an increase in Y, and vice versa. In contrast, for X–Z crosstalks with OR < 1, an increase in X would produce a decrease in Z, and vice versa. We first identified all changes of >3% in modification or charging upon each stress as well as their statistical significance, and asked whether their interdependent positions were also changing as expected (a.k.a. ‘TRUE’, Supplementary Table S5). We observed that the detected crosstalks significantly explain changes in modification and charging in all three stresses (Figure 4A, Supplementary Figure S4A). This analysis was generally robust to changes in the selected percentage threshold of 3%, with TRUE cases always exceeding FALSE ones (Supplementary Table S5). However, similar to the yeast mutants, a minority of the SLAC crosstalks did not change as expected, which reinforces the idea that detected crosstalks do not necessarily imply causality. A more detailed analysis also indicated that crosstalks with more extreme OR have generally higher percentage of TRUE cases (Supplementary Figure S4B). Moreover, TRUE cases are often dependent on the type of interconnected pair and on the amino acid family they belong to (Supplementary Figure S4C, D).

Given the near universal presence of m^1^A58 in all cytosolic tRNAs, we analyzed differences of OR between m^1^A58-charging in tRNA isodecoders and their changes upon stress. For seven out of eight 4-codon-box amino acid families (Gly, Arg, Leu, Pro, Ser, Val, Thr; Ala is the exception), we detected significantly different ORs between tRNAs with A/T versus C/G at wobble anticodon position 34 in many conditions (Figure 4B, Supplementary Figure S5A). However, directionality was dependent on the amino acid family. Because the wobble nucleotides read the third nucleotide of codons, these differences can lead to differential translational regulation of synonymous codons. As GC-ending codons appear to be more efficiently translated than AT-ending codons (Supplementary Figure S5B) and this difference is enhanced under stress (Supplementary Figure S5C) (12), these differences in m^1^A58-Charging crosstalks suggest a distinct regulation of the m^1^A58 modification and charging that unexpectedly depend on the identity of their wobble nucleotides.

To further characterize the role of m^1^A58 in translation, we compared the level of this modification between total tRNA and the polysome-associated tRNA. Surprisingly, we found that the m^1^A58 fraction is overall lower in polysome-associated tRNA than those in the total tRNA. In-depth analysis revealed, however, that this anti-selection by polysomes only occurs for tRNAs with OR > 1, but not for tRNAs with OR < 1 between m^1^A58 and charging (Figure 4C). These results are consistent with polysome selectively enriching m^1^A58-hypomodified tRNAs that are also not charged. On the other hand, the relative charging levels between polysome-associated and total RNA are about the same for tRNAs with OR > 1 and OR < 1 (Supplementary Figure S5D), which is consistent with m^1^A58-hypomodified tRNAs losing their charging (i.e. synthesizing the peptide bond) more slowly in translation. This selective enrichment may be useful to temporarily pause the polysome at specific codons (OR > 1 codons in Figure 4B), which sensitizes the polysome to rapid changes in stress.

In short, detected crosstalks provide a snapshot of the changes in modification and charging upon stress. Specifically, m^1^A58 modification appears anti-selected in translation, revealing m^1^A58-Charging crosstalk as a potential parameter of translational regulation.

### Modification crosstalks with tRNA fragmentation patterns

tRNA fragments (tRF) are small non-coding RNAs originated through enzymatic cleavage of tRNAs, which are implicated in many cellular processes such as cell proliferation, RNA silencing, or translational regulation (2). In the biogenesis of tRF, the modification state of tRNA molecules has been observed to play an important role (25–27). However, systematic analyses of existing crosstalks between tRNA modification and fragmentation have not been performed. We took advantage of MSR-seq that simultaneously sequences both full-length and fragmented tRNAs (12) and applied SLAC to uncover crosstalks. In particular, we binned tRNA fragments depending on their 3' end, classifying them into three groups: 30–39 nt (terminating at anticodon loop or C-loop), 40–49 nt (V-loop) and 50–59 nt (T-loop). Using SLAC, we then computed the odds ratios of certain modifications being detected more or less frequently in the fragmented versus the full-length tRNA reads.

**Figure 5.**
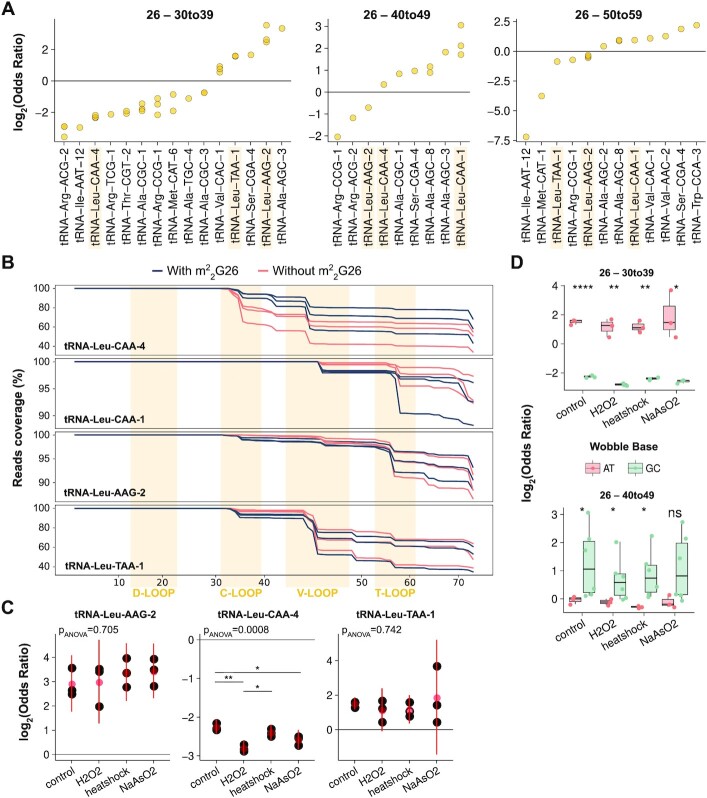
Modifications establish crosstalks with tRNA fragmentation patterns. (**A**) Distribution of OR of all significant crosstalks of m^2^_2_G26 with tRNA fragmentation at positions 30–39, 40–49 and 50–59. Each dot corresponds to individual biological replicates of the sequencing data. (**B**) Coverage of reads with and without mismatch at position 26, mapping to tRNA^Leu^(CAA)-4, tRNA^Leu^(CAA)-1, tRNA^Leu^(AAG)-2 and tRNA^Leu^(TAA)-1. The residue numbers for each tRNA is according to the tRNA nomenclature, e.g. the wobble anticodon nucleotide is always 34. tRNA transcript label is according to the genomic tRNA database (40). (**C**) Changes in OR among conditions of the 26–30 to 39 crosstalk in tRNA^Leu^(AAG)-2, tRNA^Leu^(CAA)-4 and tRNA^Leu^(TAA)-1. Black dots are individual replicates, red dots and lines indicate the mean ± SD. Significance is determined with ANOVA and post-hoc Student's *t*-test, FDR-corrected for multiple comparisons with Benjamini and Hochberg. (**D**) Differences in OR of 26–30 to 39 and 26–40 to 49 between tRNA^Leu^ isodecoders having AT versus GC at the wobble position, cognate of Leu-TTN codons. Significance is determined by a two-sided Student's *t*-test.

Among the most abundant crosstalks in control HEK293T cells (Supplementary Table S5), we found that m^2^_2_G26 is associated with fragmentation at any of the three fragment size groups (Figure 5A). tRNA^Leu^ isodecoders are frequently detected to establish m^2^_2_G26 crosstalks with multiple loops fragments (Figure 5B). In particular, for 30–39 fragmentation we observe a negative odds ratio for tRNA^Leu^(CAA)-4 which corresponds to reads containing m^2^_2_G26 being less fragmented, whereas tRNA^Leu^(TAA)-1 and tRNA^Leu^(AAG)-2 show positive odds ratios which corresponds to m^2^_2_G26 increasing fragmentation in these two tRNAs. For 40–49 fragmentation we observe a negative odds ratio for tRNA^Leu^(AAG)-2, but positive ratios for tRNA^Leu^(CAA)-4 and tRNA^Leu^(CAA)-1. For 50–59 fragmentation the odds ratios are negative for tRNA^Leu^(TAA)-1 and tRNA^Leu^(AAG)-2, but positive for tRNA^Leu^(CAA)-1. These results indicate that a single tRNA modification can have both stimulatory and inhibitory effects on tRNA fragmentation; whether stimulatory or inhibitory depends on the specific tRNA and the location of cleavage.

As tRNA fragments are known to play a role in stress response (27), we next interrogated whether these detected crosstalks were condition-dependent, focusing our analysis on the m^2^_2_G26–30 to 39 crosstalks. We observed that, while these crosstalks in some tRNAs are maintained in the unstressed and three stress conditions, for tRNA^Leu^(CAA)-4 it becomes stronger under stress (Figure 5C). Finally, we analyzed the differences between tRNA^Leu^ isodecoders, and observed that isodecoders with a GC-wobble nucleotide had negative m^2^_2_G26–30 to 39 crosstalks, while the crosstalks were positive in isodecoders with AT-wobble nucleotide (Figure 5D). In contrast, m^2^_2_G26–40 to 49 crosstalks show an opposite trend (Figure 5D), suggesting that the m^2^_2_G26 modification in tRNA^Leu^(CAA) is protective against anticodon-loop fragmentation, but induces V-loop fragmentation. Given these differences between isodecoders decoding AT-ending versus GC-ending leucine codons (Supplementary Figure S4C, D), the m^2^_2_G26 modification may also play a role in translational regulation in a way that depends on tRNA fragmentation.

Altogether, we provide evidence that single-read analysis can be extended to systematically detect crosstalks between modifications and tRNA fragmentation patterns in tRF biogenesis.

## DISCUSSION

tRNA-seq data is inherently multi-modal. While mRNA-seq is often used for quantifying transcript abundance only, tRNA-seq quantifies abundance, modification, charging and fragmentation (9,10,12,14). This work adds another aspect of tRNA-seq data which identifies networks of crosstalks between tRNA modifications, tRNA charging, and tRNA fragmentation. To make SLAC readily accessible to the community, we developed it within the open-source mim-tRNA-seq computational pipeline (https://github.com/nedialkova-lab/mim-tRNAseq) (10). The main limitation of our method requires that modifications elicit a misincorporation during the reverse transcription to generate a mutation signature. This restricts the types of modifications assessable by tRNA-seq (10,12), which are to a certain extent dependent on the tRNA-seq method of choice. However, with continuous improvements in both the experimental and computational techniques of tRNA-seq, more modification types will become accessible by including specific chemical treatments that detect otherwise silent modifications such as pseudouridine or 5-methyl-cytosine. In addition, tRNA-seq datasets intrinsically suffer from a 5' end coverage drop-off due to RT stops at some modified sites or rigid secondary structures, which leads to SLAC having a higher statistical power towards the 3' ends. Incorporation of modification-specific insertions, deletions and RT stops into the pipeline could further expand our ability to access more modification types.

So far, the study of crosstalks has been mostly limited to *ad hoc* time-course or depletion/overexpression setups followed by sequencing, mass spectrometry or NMR (28–30). For instance, m^3^C32 depended on i^6^A37 upon *Tit1* deletion (31), NMR and LC-MS of yeast tRNA^Phe^ following time-course maturation or enzyme deletions revealed modification dependencies (16), tRNA methylation was affected upon queuine depletion (32), or NSun2-mediated m^5^C methylation was protective against 5′ tRNA fragmentation (26). With the development of SLAC, we can now leverage single-read information to accurately capture tRNAome-wide associations from unperturbed and physiologically-relevant tRNA-seq datasets.

SLAC does not establish a causality in the detected crosstalks, as the existence of invariant pairs in yeast strains with modification defects suggest. Our results indicate that crosstalks identified by SLAC can be derived from multiple origins, some causal and others indirect or independent (33). Our simulation experiment indicates that SLAC can also suffer from false negatives in modification sites with > 95% mismatch rate, which is sometimes the case of tRNAs. The potential for causality or indirectness is idiosyncratic among specific tRNAs and modification sites. Even though we could not readily tell which crosstalks are causal at this time, SLAC is still useful in first identifying modification-modification, modification-charging and modification-fragmentation crosstalks that otherwise elude our current methods of detecting them.

Our systematic analysis of human tRNA crosstalks reveals that modifications and charging can be interconnected, potentially wiring a complex regulatory network. By analyzing the observed changes upon three different stresses, we support the physiological relevance of tRNA crosstalk networks in recapitulating the changes of modifications and charging. Translation has been widely studied in the context of stress response, which has been related to defects in protein homeostasis and human disease (34–36). For instance, the differential expression of tRNAs recognizing A- versus G-ending codons in polysomes, together with changes of specific tRNA modifications, leads to the selective enhancement of stress-dependent transcripts with a biased codon usage towards G/C-ending-codons (12). Here we provide evidence that tRNA crosstalks play a role in the stress response, and that m^1^A58-Charging crosstalk can be differently wired between isodecoders decoding AT- versus GC-ending codons.

The tRNA m^1^A58 is among the most interconnected modifications with m^1^A58-m^2^_2_G26 and m^1^A58-m^1^G37 as abundant crosstalks in the human tRNAome, and also constitutes the most tissue-specific modification in mice. These results indicate that m^1^A58 showcases a previously underappreciated regulatory potential. In this context, we observe that m^1^A58-modification levels for some tRNAs are lower in polysomes compared to total tRNA, however, this result is only for tRNAs with a positive m^1^A58-charging crosstalk. This result is consistent with ribosome subtlely enriching tRNAs lacking m^1^A58 modification which are also not charged. Presumably, this type of selection by the polysome may recapitulate an enrichment of uncharged and m^1^A58-hypomodified tRNA in the A-site for translational pausing.

Beyond translation, tRNAs can be enzymatically cleaved and give rise to tRNA fragments which are involved in many processes such as stress response, cancer, aging, or development (2,37). By extending our single-read tRNA analysis to tRFs, we identify specific tRNA modifications that either correlate or anti-correlate with fragment biogenesis, which recapitulates previously observed associations (12). In particular, we detect m^2^_2_G26 as a crucial parameter for tRNA^Leu^ fragmentation; this modification can be either protective or inductive for fragmentation depending on the specific isodecoder and the location of the cleavage site. The magnitude of these crosstalks can also be modulated under stress.

The networks of crosstalks that we identified are diverse at the tRNA isodecoder level. These networks are dynamic in stress response and distinct in mouse tissues. As such, the tRNA crosstalk networks are, at first approach, resistant to satisfying and simple rules—a theme in tRNA biology harkening back to deciphering protein-RNA interactions between aminoacyl-synthetases and specific tRNA substrates (38). There is likely a complex grammar that we have just started to decipher, that introduces many new questions. What mechanisms give rise to crosstalks: tRNA biogenesis, tRNA synthetases, writer and eraser enzymes, nuclear export and re-import? How do these crosstalks affect translation in each tRNA context? On what timescales are these networks wired and rewired; does rewiring require tRNA turnover? Answering these questions will benefit from the characteristically high sequencing depth of many tRNA-seq datasets and their potential to detect for the first time tRNA crosstalks *in vivo*, such as the reported m^1^A58-m^1^G9 crosstalk of tRNA^Glu^(TTC)-1 detected in liver and heart but not in other mouse tissues. Our work opens a new avenue to study crosstalks in physiologically-relevant conditions or diseases such as cancer that are known to alter tRNAomes and ultimately translation (1,7,39).

## DATA AVAILABILITY

The code used in this study is available at GitHub (https://github.com/hexavier/tRNA_crosstalks), and the single-read analysis software is available within the open-source mim-tRNA-seq computational pipeline (https://github.com/hexavier/mim-tRNAseq). All datasets and code generated or analyzed during this study are available at Figshare (https://doi.org/10.6084/m9.figshare.c.6311601).

## Supplementary Material

gkac1185_Supplemental_Files
