# Supplementary material for: Single-read tRNA-seq analysis reveals coordination of tRNA modification and aminoacylation and fragmentation
Source: Nucleic Acids Res. 2022 Dec 20;51(3):e17. doi: 10.1093/nar/gkac1185 (PMC9943672; doi:10.1093/nar/gkac1185)
Supplement: gkac1185_Supplemental_Files [file gkac1185_supplemental_files.zip › NAR_SupData.pdf]

SUPPLEMENTARY FIGURES

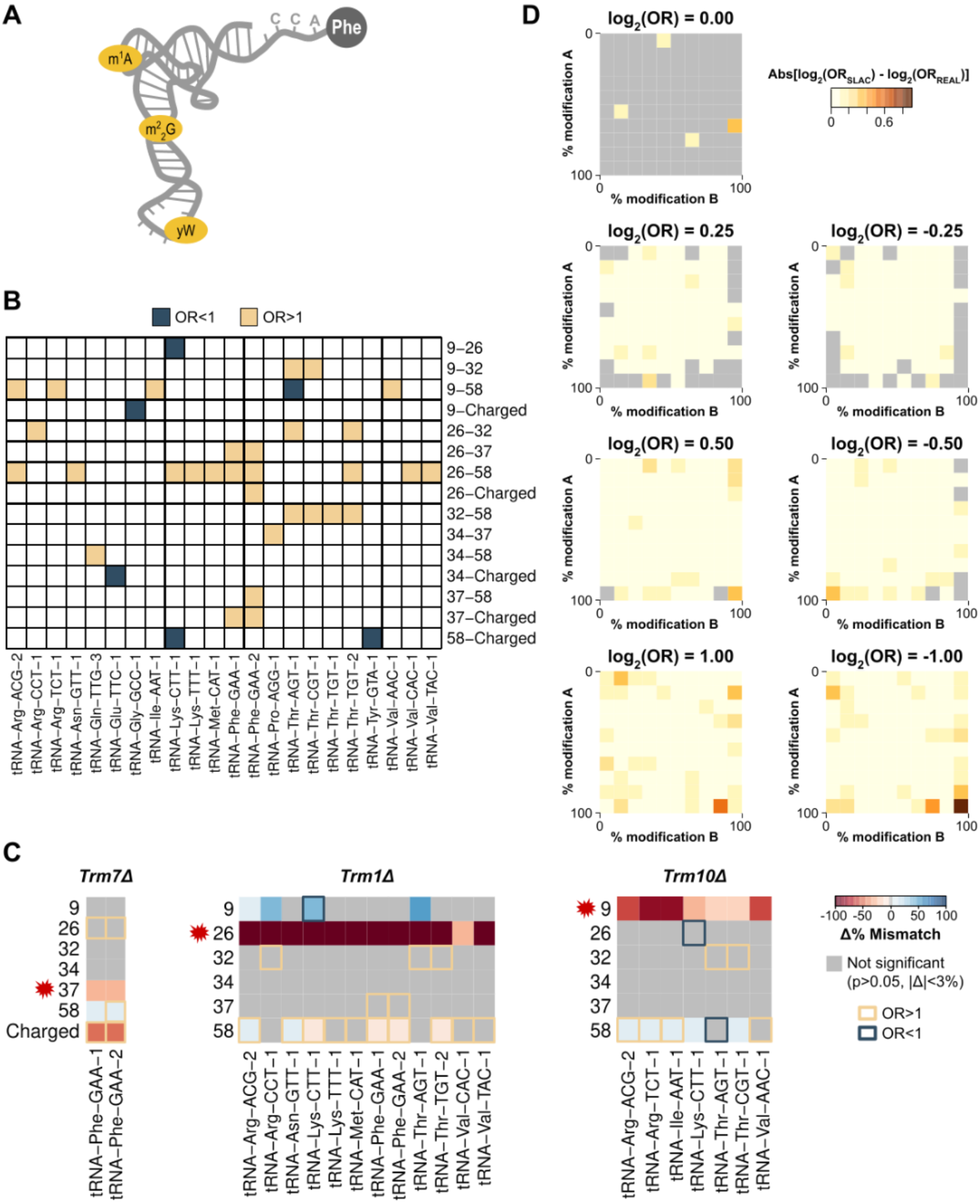

Figure S1. Single-read tRNA-seq analysis reveals known and new crosstalks in yeast tRNA. (A) Three-dimensional diagram of detected modifications in yeast $tRNA^{Phe}$. (B) Heatmap of all significant crosstalks in at least two replicates of WT yeast tRNAs. The color represents the OR of each pair. The residue numbers for each tRNA is according to the tRNA nomenclature, e.g. the wobble anticodon nucleotide is always 34. tRNA transcript label is according to the genomic tRNA database. Known modifications include m^1G9, m^3C32, m^2_2G26, m^1G37, and m^1A58. (C) Changes in mismatch and aminoacylation levels upon deletion of three modification enzymes (*Trm7* for yW37 of $tRNA^{Phe}$, *Trm1*

for m^2_2G26, *Trm10* for m^1G9, position indicated by a red star). Significant changes include positions with p-value<0.05 by chi-square tests after FDR correction with Benjamini & Hochberg and >3% absolute difference. All positions with a detected crosstalk in panel B are shown, with orange and blue squares indicating the corresponding crosstalk OR. *Trm1Δ* and *Trm10Δ* libraries were not periodate oxidized and therefore aminoacylation levels are not detectable. (D) Absolute errors (Abs[log2(OR_{SLAC}) - log2(OR_{REAL})]) of simulated reads with two modification sites, with varying percentages of modifications and different ORs. Gray cells correspond to reported non-significant crosstalks.

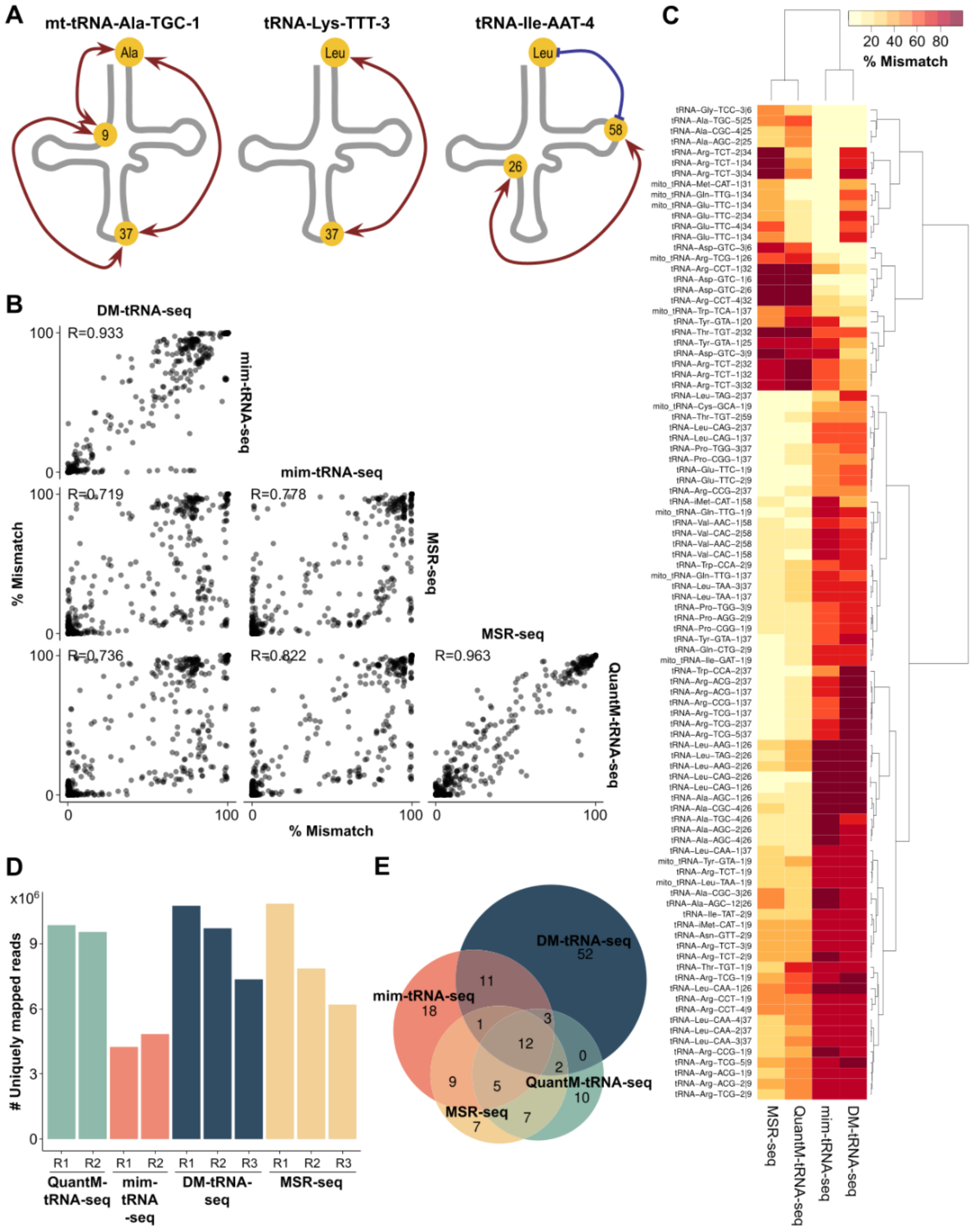

Figure S2. Comparing tRNA crosstalks among different tRNA-seq methods. (A) Significant crosstalks detected in at least two of the 3 biological replicates of mt-tRNA^{Ala}(TGC)-1, tRNA^{Lys}(TTT)-3, and tRNA^{Ile}(AAT)-4. (B) Pearson correlations of mismatch percentages of HEK293T cells by mim-tRNA-seq, DM-tRNA-seq, QuantM-tRNA-seq, and MSR-seq, including all positions >5% in at least one method. Replicates of each method are averaged. (C) Mismatch patterns of all positions with SD > 20% among the four tRNA-seq methods. Modifications and methods are hierarchically clustered using euclidean distances and the Ward's minimum variance method. (D) Uniquely mapping reads to tRNA genes in each sample of the four tRNA-seq methods. (E) Venn diagram of significant crosstalks in all replicates of HEK293T cells by 4 tRNA-seq methods (mim-tRNA-seq, DM-tRNA-seq,

QuantM-tRNA-seq, and MSR-seq). Only the 248 consensus modification sites (Fig. 2E) between these methods are included.

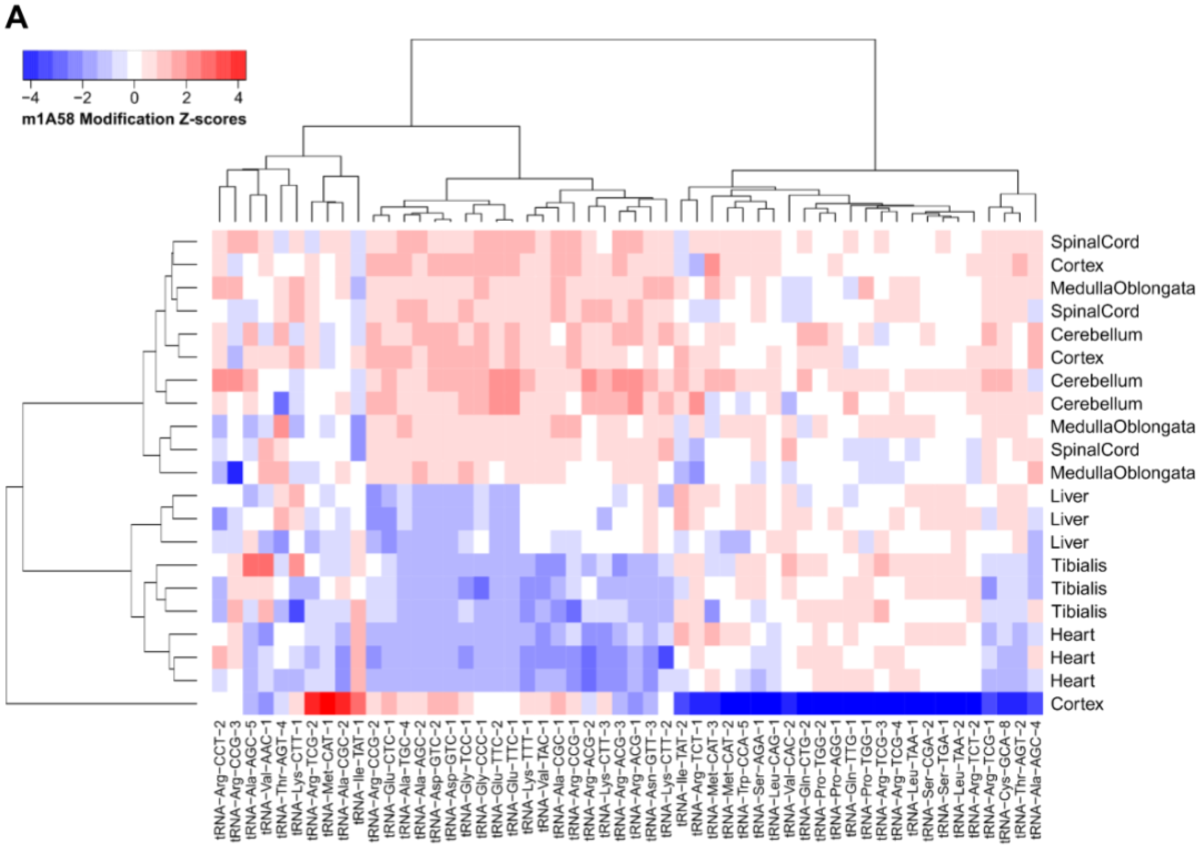

Figure S3. Tissue-specificity of modification m^{1}A58 and crosstalks across mouse tissues. (A) Standardized m^{1}A58 modification levels across tissues. Isodecoders and tissues are hierarchically clustered using euclidean distances and the Ward's minimum variance method.

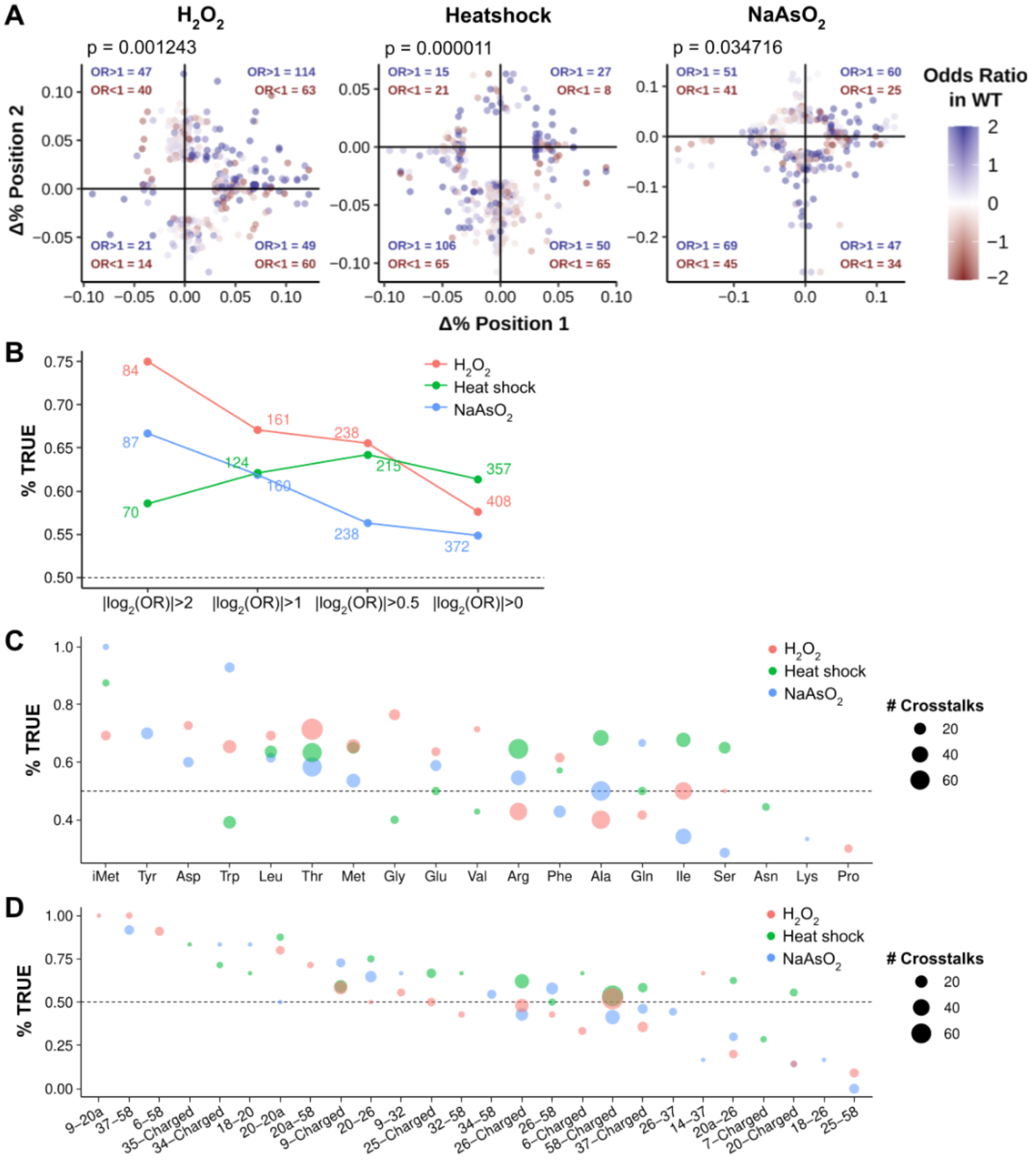

Figure S4. Crosstalks recapitulate modification and charging changes upon stress. (A) Changes in charging and modifications upon H_2O_2, heat shock and $NaAsO_2$ stress, shown as the difference between individual stressed replicates and the average of control HEK293T. All significant crosstalks among pair changes of >3% in mismatch of read alignment are included. A one-sided binomial test is used to determine whether observed frequency of successes [e.g. for heat shock: (106+27+21+65)/357=0.61] is higher than the null model p=0.5. (B) Among all changes depicted in Fig. 4A and Fig. S4A, percentage of crosstalks behaving as expected by OR (TRUE) taking subsets of pairs over a certain $|log_2(OR)|$. Numbers indicate the total number of crosstalks of each subset from which the percentage is computed. (C) Percentage of crosstalks behaving as expected by OR (TRUE) grouping them by amino acid families. (D) Percentage of crosstalks behaving as expected by OR (TRUE) grouping them by pairs.

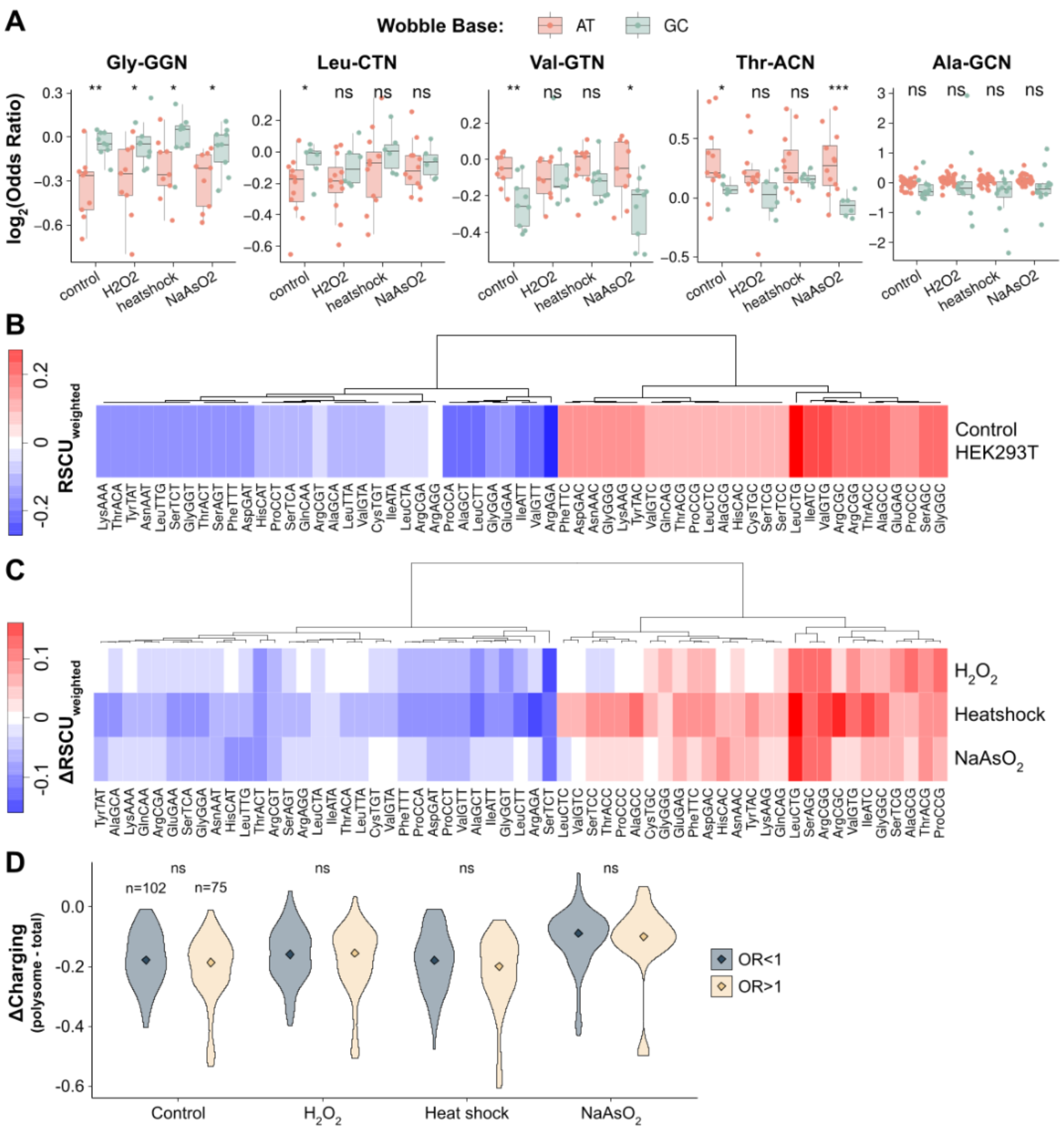

Figure S5. Effect of crosstalks in translational regulation. (A) Differences in OR of 58-Charged between isodecoders having AT vs GC at the wobble position of the 4 codon box readers of $tRNA^{Gly}$, $tRNA^{Leu}$, $tRNA^{Val}$, $tRNA^{Thr}$, and $tRNA^{Ala}$. Significance is determined by two-sided Student's t-test. (B) Relative Synonymous Codon Usage (RSCU) weighted by the translational efficiency (TE) of the human translatome in unstressed HEK293T cells, computed as the ratio between polysome TPMs versus total RNA TPMs. Codons are hierarchically clustered using euclidean distances and the Ward's minimum variance method. (C) Difference of the RSCU weighted by the TE between stress conditions and control HEK293T cells, computed as the ratio between polysome TPMs versus total RNA TPMs. Codons are hierarchically clustered using euclidean distances and the Ward's minimum variance method. (D) Differences in charging between polysome vs total tRNAs, among all isodecoders with a significant crosstalk in at least one replicate. Changes between tRNAs with OR>1 and OR<1 are detected by two-tailed Wilcoxon rank-sum test. tRNA charging levels in the polysome are generally lower than total RNA because of the uncharged tRNA in the E-site of the ribosome.

SUPPLEMENTARY TABLES

Table S1. Modifications detectable by tRNA-seq. Collection of known modifications and their corresponding sites in cytoplasmic human tRNAs (Suzuki, 2021).

Abbreviation	Modification[a]	Positions	Mismatch in tRNA alignment[b]
ac4C	N4-acetylcytidine	12	
acp3U	3-(3-amino-3-carboxypropyl)uridine	20,20a	Yes
Cm	2′-O-methylcytidine	4,32,34	
D	Dihydrouridine	16,17,20,20a,47	
f5Cm	5-formyl-2′-O-methylcytidine	34	
galQ	Galactosyl-queuosine	34	
Gm	2′-O-methylguanosine	18,34,39	
hm5C	5-hydroxymethylcytidine	34	
hm5Cm	2′-O-methyl-5-hydroxymethylcytidine	34	
I	Inosine	34	Yes
i6A	N6-isopentenyladenosine	37	
m1A	1-methyladenosine	9,14,58	Yes
m1G	1-methylguanosine	9,37	Yes
m1I	1-methylinosine	37	Yes
m2,2G	N2,N2-dimethylguanosine	26,27	Yes
m2G	N2-methylguanosine	6,7,10,26	Yes
m3C	3-methylcytidine	32,e2	Yes
m5C	5-methylcytidine	38,48,49,50,72	
m5U	5-methyluridine	54	
m5Um	2′-O-methyl-5-methyluridine	54	
m6t6A	N6-methyl-N6-threonylcarbamoyladenosine	37	Yes
m7G	7-methylguanosine	46	
manQ	Mannosyl-queuosine	34	
mchm5U (R)	5-(carboxyhydroxymethyl)uridine methyl ester	34	
mchm5U (S)	5-(carboxyhydroxymethyl)uridine methyl ester	34	
mcm5s2U	5-methoxycarbonylmethyl-2-thiouridine	34	
mcm5U	5-methoxycarbonylmethyluridine	34	
mcm5Um	5-(carboxyhydroxymethyl)-2′-O-methyluridine	34	
mpG	5′-methylphosphoguanosine	-1	
ms2t6A	2-methylthio-N6-threonylcarbamoyladenosine	37	Yes
ncm5U	5-carbamoylmethyluridine	34	
o2yW	Peroxywybutosine	37	Yes
OHyW	Hydroxywybutosine	37	
Q	Queuosine	34	
t6A	N6-threonylcarbamoyladenosine	37	
Um	2′-O-methyluridine	4,32,39,44,e12	
Ψ	Pseudouridine	8,13,20b,27,28,30,31,32,34,35,38,39,e12,e1,50,54,55,72	
Ψm	2′-O-methylpseudouridine	32,39	

a. The frequency of each modification type is highly uneven among human tRNAs. For example, galQ is present only in cytosolic $tRNA^{Tyr}$, whereas m^1A58 is present in all cytosolic tRNAs.

b. Detectable modifications through mutation signatures in data analysis from tRNA-seq libraries using both SuperScript IV RT in QuantM-seq and MSR-seq and TGIRT in DM-tRNA-seq and mim-tRNA-seq.

Table S2. Crosstalks in *S. cerevisiae*. (A) All analyzed crosstalks of mim-tRNA-seq (GSE152621). (B) Significant crosstalks of mim-tRNA-seq (GSE152621). (C) tRNA charging of mim-tRNA-seq (GSE152621). (D) tRNA modifications of mim-tRNA-seq (GSE152621). (E) Differentially modified and charged positions in mutant yeast strains. (F) tRNA modifications of simulated reads. (G) All analyzed crosstalks of simulated reads.

Table S3. Crosstalks in HEK293T. (A) All analyzed crosstalks of DM-tRNA-seq (GSE97259). (B) Significant crosstalks of DM-tRNA-seq (GSE97259). (C) All analyzed crosstalks of control HEK293T by MSR-seq, mim-tRNA-seq, QuantM-tRNA-seq, DM-tRNA-seq. (D) Significant crosstalks of control HEK293T by MSR-seq, mim-tRNA-seq, QuantM-tRNA-seq, DM-tRNA-seq. (E) tRNA modifications of control HEK293T by MSR-seq, mim-tRNA-seq, QuantM-tRNA-seq, DM-tRNA-seq. (F) Overlap of significant crosstalks by MSR-seq, mim-tRNA-seq, QuantM-tRNA-seq, DM-tRNA-seq.

Table S4. Crosstalks in mouse tissues. (A) All analyzed crosstalks of QuantM-tRNA-seq (GSE141436). (B) Significant crosstalks of QuantM-tRNA-seq (GSE141436). (C) tRNA modifications of QuantM-tRNA-seq (GSE141436).

Table S5. Crosstalks in MSR-seq. (A) All analyzed crosstalks of MSR-seq (GSE198441). (B) Significant crosstalks of MSR-seq (GSE198441). (C) tRNA charging of MSR-seq (GSE198441). (D) tRNA modifications of MSR-seq (GSE198441). (E) tRNA abundance of MSR-seq (GSE198441). (F) Differentially modified and charged positions under stress. (G) Binomial tests of HEK293T changes under stress. (H) All analyzed tRNA fragmentation crosstalks of MSR-seq. (I) Significant tRNA fragmentation crosstalks of MSR-seq.
